# The emergency discharge of sewage to the Bay of Gdańsk as a source of bacterial enrichment in coastal air

**DOI:** 10.1038/s41598-021-00390-8

**Published:** 2021-10-25

**Authors:** Małgorzata Michalska, Katarzyna Zorena, Roman Marks, Piotr Wąż

**Affiliations:** 1 Department of Immunobiology and Environment Microbiology, Faculty of Health Sciences with Institute of Maritime and Tropical Medicine Medical University of Gdańsk, ul. Dębinki 7, 80-211 Gdańsk, Poland; 2 Institute of Marine and Environmental Sciences, University of Szczecin, ul. Mickiewicza 16, 70-383 Szczecin, Poland; 3 Department of Nuclear Medicine, Faculty of Health Sciences with Institute of Maritime and Tropical Medicine, Medical University of Gdańsk, ul. Dębinki 7, 80-211 Gdańsk, Poland

**Keywords:** Environmental sciences, Hydrology, Natural hazards

## Abstract

The purpose of this research was to study the presence of potential pathogenic bacteria in the seawater and air in five coastal towns (Hel, Puck, Gdynia, Sopot, Gdańsk-Brzeźno) as well as the enrichment of bacteria from the seawater into the coastal air after an emergency discharge of sewage into the Bay of Gdańsk. A total of 594 samples of air and seawater were collected in the coastal zone between spring and summer (between 2014 and 2018). Air samples were collected using the impact method with a SAS Super ISO 100. The multivariate analysis, conducted using contingency tables, showed a statistically significant variation between the concentration of coliforms, psychrophilic and mesophilic bacteria in the seawater microlayer and air in 2018, after an emergency discharge of sewage into the Bay of Gdańsk, compared to 2014–2017. Moreover, we detected a marine aerosol enrichment in psychrophilic, mesophilic bacteria, coliforms and *Escherichia coli****.*** We also showed a statistically significant relationship between the total concentration of bacteria and humidity, air temperature, speed and wind direction. This increased concentration of bacteria in the seawater and coastal air, and the high factor of air enrichment with bacteria maybe associated with the emergency discharge of wastewater into the Bay of Gdańsk. Therefore, it is suggested that in the event of a malfunction of a sewage treatment plant, as well as after floods or sudden rainfall, the public should be informed about the sanitary and epidemiological status of the coastal waters and be recommended to limit their use of coastal leisure areas.

## Introduction

Physicochemical and microbiological pollution of coastal waters is an important issue in low and middle income countries (LMICs)^1^. Coastal aerosols can have a major influence on the planet’s ecology and climate, and have been associated with a suite of human health problems^2–5^. The presence of bacteria, mould spores and their metabolic products (e.g. endotoxins or mycotoxins) in sea spray aerosols (SSA) may be one of the main factors influencing human infections^6,7^. The latest research shows that seawater pollution with potentially pathogenic bacteria belonging to faecal streptococci (Enterococci), coliforms or *Escherichia coli* is a significant threat to human health^7–10^. The bacteria and components of their cell walls (e.g. D-glucans) can cause gastrointestinal and respiratory tract disorders or skin allergies and, as a consequence, contribute to many illnesses^11–16^. Faecal coliforms live primarily in the human digestive tract. Their presence in the natural environment is a result of municipal wastewater discharges and runoffs from urban and agricultural areas into surface waters^17–22^. Many years of research by ourselves and other researchers have showed that despite a very high reduction in the concentration of bacteria during the treatment process, treated sewage still contains more than 10^4^ CFU/100 ml of faecal coliforms^17,23–25^. It is known thatheavy rainfall or floods contribute to a higher concentration of faecal bacteria in coastal seawaters^26–28^. Another problem are sewage pumping station failures. For example, due to a malfunction at the Mishref Pumping Station (Kuwait), around 150,000 m^3^/day of raw sewage was being discharged directly into the bay for a period of 3 years (2009–2012). This led to a bacteriological contamination of a coastal section of about 20 km in the Kuwait Bay waters^29^.

The quality of seawater is assessed not only by examining the number of intestinal bacteria, but also the total number of psychrophilic and mesophilic bacteria, *Pseudomonas aeruginosa* and *Staphylococcus aureus*^30–34^. For example, the number of mesophilic bacteria indicates the presence of pathogenic and potentially pathogenic microorganisms in the water. On the other hand, the number of psychrophilic bacteria indicates the organic matter content in the water^30,31^. *P. aeruginosa* is able to survive during long periods without nutrients in a dormant state, and significantly longer than the two other bacterial pathogens, *E. coli* and *S. aureus*^32–34^. *S. aureus*, on the other hand, is an opportunistic pathogen and is known to survive at higher salinity^35^. From May 15th to 18th (for 3 days) 2018, there were emergency discharges of raw sewage into the Motława River, which then went into the Bay of Gdańsk. As a result, 2300 m^3^ of untreated municipal wastewater were discharged into the Bay of Gdańsk per hour.

In our preliminary study, we presented the results about the increase of potentially pathogenic mesophilic bacteria in the seawater and coastal air in 2018 after an emergency discharge of raw sewage from the Gdańsk–Wschód wastewater plant into the Bay of Gdańsk^36^. The aim of the present study was to compare the bacterial concentration in seawater and air in five coastal towns before (2014–2017) and after (2018) an emergency discharge of sewage into the Bay of Gdańsk. Moreover, we assessed the enrichment of bacteria of the coastal air, over the Bay of Gdańsk, from the surface microlayer (SML) by the eruption of rising bubbles, based on the data obtained in Hel, Puck, Gdynia, Sopot and Gdańsk-Brzeźno.

The SML is a unique biological, physical and chemical environment at the interface between the atmosphere and the hydrosphere. The SML contains elevated concentrations of heterotrophic and autotrophic microorganisms including bacteria, cyanobacteria, flagellates and algae^38^. These microorganisms impact air quality through transmission of allergens and pathogens and play a key role in global biogeochemical and climate regulation processes^1,4,5,37,38^. Very few studies have investigated the enrichment of air with microorganisms after storms and heavy rains^39^, and to our knowledge, there are currently no studies linking emergency discharges of raw sewage to microbial air enrichment. Therefore, the failure of the sewage pumping station in the Bay of Gdańsk offered a unique opportunity to investigate the effects of sewage discharges on bacterial enrichment of coastal air.

## Results

### Analysis of bacteria in the sea-surface microlayer in the 5 coastal towns of the Bay of Gdańsk in 2018 and during 2014–2017

The concentration of psychrophilic and mesophilic bacteria in the sea-surface microlayer samples was higher in 2018 than in 2014–2017 at the seaside towns of Hel, Gdynia, Sopot and Gdańsk-Brzeźno. Using the non-parametric Friedman rank sum test, we demonstrated a statistically significant difference between the mean concentration of psychrophilic and mesophilic bacteria in the 5 seaside towns before and after the discharge (χ^2^ = 5, df = 1, p = 0.025). The mean, median, and standard deviation values for the concentration of psychrophilic and mesophilic bacteria are presented in Table 1.

**Table 1:** The mean and standard deviation of concentration of psychrophilic and mesophilic bacteria (CFU/1 ml) in sea-surface microlayer samples collected in the Bay of Gdańsk in 2018 versus 2014–2017.

Location	Psychrophilic bacteria (CFU/1 ml)	Psychrophilic bacteria (CFU/1 ml)	Fold change	Mesophilic bacteria (CFU/1 ml)	Mesophilic bacteria (CFU/1 ml)	Fold change
	2018*	2014–2017	2018 vs 2014–2017	2018*	2014–2017	2018 vs 2014–2017
Hel	3.34 × 10^5^ ± 3.115 × 10^5^	1.019 × 10^4^ ± 1.351 × 10^4^; 6 × 10^3^ (200–4.8 × 10^3^)^b^	33	3.115 × 10^5^ ± 3.507 × 10^5^; 3.9 × 10^5^ (4 × 10^3^–6.3 × 10^5^)^b^	2.715 × 10^3^ ± 2.719 × 10^3^; 1.8 × 10^3^ (100–8.9 × 10^3^)^b^	114
Puck	5.433 × 10^3^ ± 8.62 × 10^2^	4.218 × 10^3^ ± 1.624 × 10^3^	1,3	3.967 × 10^3^ ± 6.11 × 10^2^	2.2 × 10^3^ ± 2.1 × 10^3^	2
Gdynia	1.508 × 10^4^ ± 1.326 × 10^3^	5.232 × 10^3^ ± 1.315 × 10^3^	3	7.008 × 10^3^ ± 4.195 × 10^3^	3.110 × 10^3^ ± 1.316 × 10^3^	2
Sopot	1.123 × 10^4^ ± 6.621 × 10^3^	4.617 × 10^3^ ± 1.213 × 10^3^	2,4	9.146 × 10^3^ ± 5.606 × 10^3^	2.073 × 10^3^ ± 8.88 × 10^2^	4,4
Gd.-Brzeźno ^a^	2.411 × 10^4^ ± 4.731 × 10^4^; 1 × 10^4^ (4.5 × 10^3^–1.5 × 10^5^)^b^	4.292 × 10^3^ ± 9.95 × 10^2^	6	1.585 × 10^4^ ± 2.423 × 10^4^; 1 × 10^4^ (4.2 × 10^3^–8 × 10^4^)^b^	3.787 × 10^3^ ± 5.85 × 10^2^	4,2
p-value^c^	0.025			0.025		

^a^Gd.-Brzeźno—it is an abbreviation of the town name Gdańsk-Brzeźno, ^b^in case of a higher standard deviation than the mean, the median and range (min–max) was calculated, ^c^Friedman test, α-0.05, *The results marked in grey colour have been published in preliminary study^36^.

In 2018 the concentration of coliforms in the SML collected from the Bay of Gdańsk, in Puck, Sopot and Gdańsk-Brzeźno, was higher compared to 2014–2017 but not statistically significant (χ^2^ = 1.8, df = 1 p = 0.18). The concentration of *E. coli* in SML samples was higher in 2018 than in 2014–2017 at the Puck and Gdańsk-Brzeźno seaside towns, but nostatistical significance was found (χ^2^ = 0.2, df = 1, p = 0.655) (Table 2).

**Table 2:** The mean and standard deviation of concentration of coliforms and *E*. *coli* (CFU/100 ml) in sea-surface microlayer samples collected in the Bay of Gdansk in 2018 versus 2014–2017.

Location	Coliform bacteria (CFU/100 ml)	Coliform bacteria (CFU/100 ml)	Fold change	*E. coli* (CFU/100 ml)	*E. coli* (CFU/100 ml)	Fold change
	2018*	2014–2017	2018 vs 2014–2017	2018*	2014–2017	2018 vs 2014–2017
Hel	2.805 × 10^3^ ± 2.8 × 10^3^ 2.31 × 10^3^ (4 × 10^2^–6.2 × 10^3^)^b^	3.587 × 10^3^ ± 2.497 × 10^3^	–	2.06 × 10^2^ ± 3.96 × 10^2^ 12 (0–8 × 10^2^)^b^	4.46 × 10^2^ ± 5.42 × 10^2^; 2.3 × 10^2^ (0–1.8 × 10^3^)^b^	–
Puck	1.157 × 10^3^ ± 1.69 × 10^2^	3.73 × 10^2^ ± 2.97 × 10^2^	3	2.23 × 10^2^ ± 1.2 × 10^1^	1.9 × 10^1^ ± 8	11
Gdynia	2.5 × 10^3^ ± 3.81 × 10^2^	1.612 × 10^3^ ± 9.74 × 10^2^ 9.74 × 10^2^ (2.3 × 10^2^–2.3 × 10^3^)^b^	1,5	10 ± 7	5.61 × 10^2^ ± 9.78 × 10^2^ 9.78 × 10^2^ (23–2.3 × 10^3^)^b^	–
Sopot	3.280 × 10^4^ ± 8.006 × 10^4^ 1.265 × 10^3^ (2.3 × 10^2^–2.3 × 10^5^)^b^	5.7 × 10^2^ ± 8.7 × 10^1^	57	1.39 × 10^2^ ± 1.79 × 10^2^ 72 (0–5.1 × 10^2^)^b^	3.6 × 10^2^ ± 2.25 × 10^2^	–
Gd.-Brzeźno ^a^	5.439 × 10^3^ ± 7.452 × 10^3^ 2.3 × 10^3^ (2.3 × 10^2^–2.4 × 10^4^)^b^	2.13 × 10^2^ ± 1.5 × 10^1^	26	2.11 × 10^2^ ± 2.28 × 10^2^ 1.3 × 10^2^ (0–7 × 10^2^)^b^	1.89 × 10^2^ ± 3.6 × 10^1^	1
p-value^c^	0.180			0.655		

^a^Gd.-Brzeźno—it is an abbreviation of the town name Gdańsk-Brzeźno, ^b^in case of a higher standard deviation than the mean, the median and range (min–max) was calculated, ^c^Friedman test, α-0.05, *The results marked in grey colour have been published in preliminary study^36^.

The concentrations of *P. aeruginosa* and *S. aureus* detected in the SML were higher in 2018 than in 2014–2017, in Hel, Sopot and Gdańsk-Brzeźno, but not statistically significant (χ^2^ = 1, df = 1, p = 0.317). The results are presented in Table 3.

**Table 3:** The mean and standard deviation of concentration of *P. aeruginosa* and *S. aureus* (CFU/100 ml) in sea-surface microlayer water collected from the Bay of Gdansk in 2018 versus 2014-–2017.

Location	*P. aeruginosa* (CFU/100 ml)	*P. aeruginosa* (CFU/100 ml)	Fold change	*S. aureus* (CFU/100 ml)	*S. aureus* (CFU/100 ml)	Fold change
	2018	2014–2017	2018 vs 2014–2017	2018	2014–2017	2018 vs 2014–2017
Hel	1.7 × 10^3^ ± 2 × 10^3^; 16 (0–3.6 × 10^3^)^b^	1.31 × 10^2^ ± 3.01 × 10^2^; 0 (0–9 × 10^2^)^b^	13	1.5 × 10^2^ ± 3 × 10^2^; 0 (0–6 × 10^2^)^b^	1.5 × 10^1^ ± 5.5 × 10^1^; 0 (0–2 × 10^2^)^b^	10
Puck	0.00	0.00	–	1.67 × 10^2^ ± 2.89 × 10^2^; 0 (0–5 × 10^2^)^b^	1.5 × 10^2^ ± 1.29 × 10^2^	1
Gdynia	0.00	1 × 10^2^ ± 2.24 × 10^2^; 0 (0–5 × 10^2^)^b^	–	1.2 × 10^2^ ± 1.3 × 10^2^; 1 × 10^2^ (0–3 × 10^2^)^b^	6 × 10^1^ ± 8.9 × 10^1^ 0 (0–2 × 10^2^)^b^	20
Sopot	4.75 × 10^2^ ± 7.65 × 10^2^; 0 (0–2.1 × 10^3^)	0.00	5	1.63 × 10^2^ ± 2.26 × 10^2^; 0 (0–5 × 10^2^)^b^	1.33 × 10^2^ ± 2.32 × 10^2^; 0 (0–4 × 10^2^)^b^	1
Gd.-Brzeźnoa	4.89 × 10^2^ ± 8.01 × 10^2^; 0 (0–2.2 × 10^3^)^b^	0.00	5	1.67 × 10^2^ ± 3.08 × 10^2^; 0 (0–8 × 10^2^)^b^	1 × 10^2^ ± 1.73 × 10^2^; 0 (0–3 × 10^2^)^b^	2
p-value^c^	0.317			0.317		

^a^Gd.-Brzeźno—it is an abbreviation of the town name Gdańsk-Brzeźno, ^b^in case of a higher standard deviation than the mean, the median and range (min–max) was calculated, ^c^Friedman test, α-0.05.

### The relationship between the concentration of bacteria detected in the SML samples collected over the Bay of Gdańsk in the 5 coastal towns in 2018 and the 2014–2017 period

**Figure 1:**
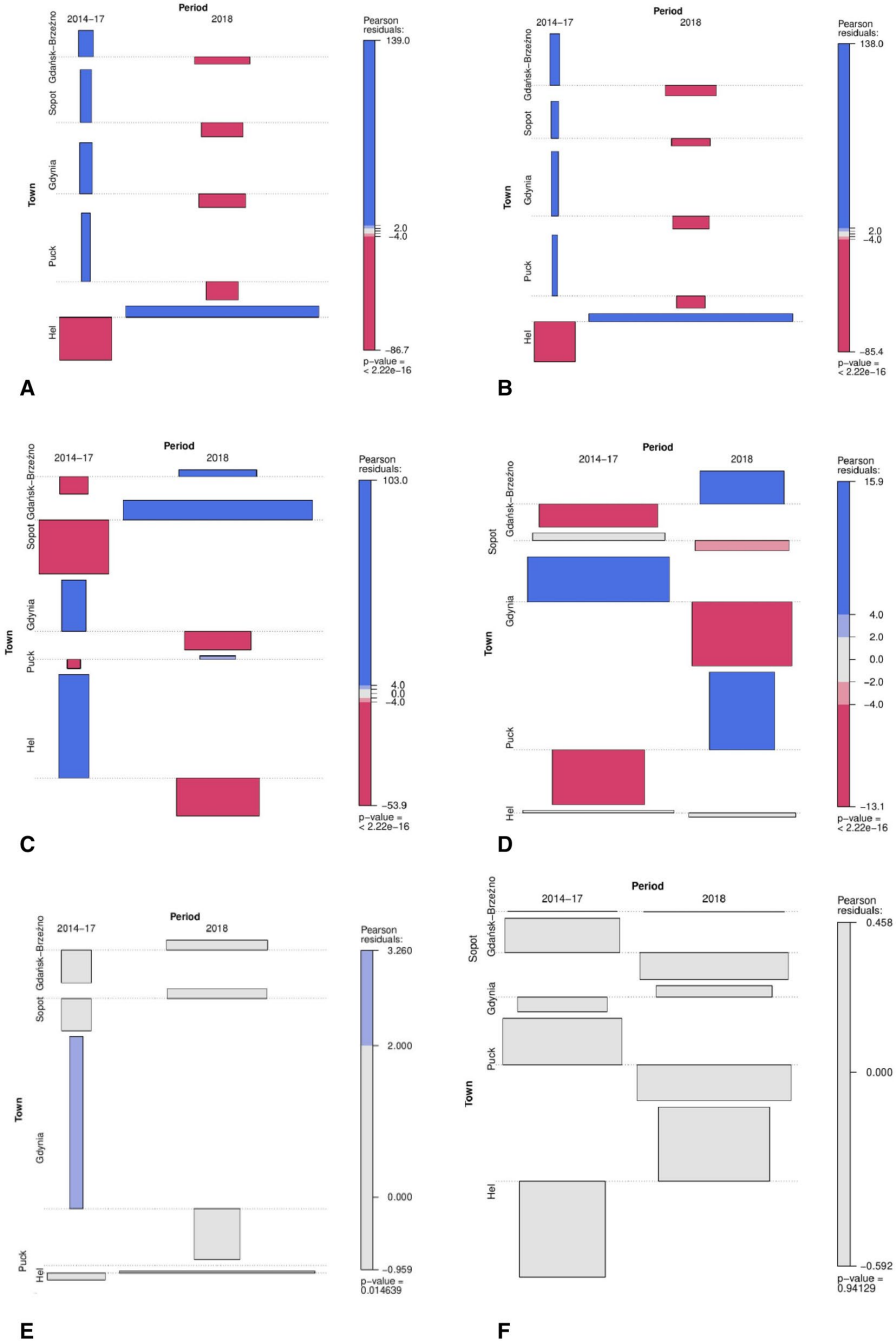
The association of the contingency table with the mean quantity of psychrophilic (**A**) and mesophilic bacteria (**B**), coliform (**C**) and *E. coli* (**D**) bacteria, *P. aeruginosa* (**E**) and *S.aureus* (**F**) bacteria detected in the SSA samples collected from the Bay of Gdańsk in Hel, Puck, Gdynia, Sopot and Gdańsk-Brzeźno and the time period for the sample collection (2014–2017 and 2018). The blue colour indicates that the observed value is higher than expected if the data was random. The red colour specifies that the observed value is expected value and the grey colour indicates that the observed value is close to the expected.

A Pearson chi-squared test of independence showed that during 2014–2017 in Hel, there was a lower concentration of psychrophilic and mesophilic bacteria than expected. In 2018, on the other hand, a higher concentration of psychrophilic (χ^2^ = 55,643, df = 4, p = < 2.22 × 10^−16^) and mesophilic bacteria (χ^2^ = 63,928, df = 4, p = < 2.22 × 10^−16^) was detected in Hel compared to the expected value according to the use of the independence test (Fig. 1A, B).

In 2018, in Sopot and Gdańsk-Brzeźno, a higher than expected concentration of coliforms was recorded, while in 2014–2017 it was lower. The result was statistically significant (χ^2^ = 18,656, df = 4, p = < 2.22 × 10^−16^). Also, in 2018, in Puck and Gdańsk-Brzeźno, the concentration of *E. coli* was higher than expected, while in 2014–2017 it was lower (χ^2^ = 709.96, df = 4, p = < 2.22 × 10^−16^) (Fig. 1C, D).

The results during 2014–2017 and in 2018 showed that in Hel, Sopot and Gdańsk-Brzeźno the mean concentration of *P. aeruginosa* and *S. aureus* was very close to the expected value, according to the use of the independence test. The result was statistically significant for the mean concentration of *P. aeruginosa* (χ^2^ = 12.7, df = 4, p = 0.015). However, no statistical significance was demonstrated when comparing the average concentration of *S. aureus* bacteria in the water in 2014–2017 vs 2018 (χ^2^ = 0.778, df = 4, p = 0.941) (Fig. 1E, F).

### Analysis of bacteria in the air in the 5 coastal towns of the Bay of Gdańsk in 2018 compared to 2014-2017

The concentrations of psychrophilic and mesophilic bacteria in the sea spray aerosol (SSA), from Gdynia, Sopot and Gdańsk-Brzeźno, were significantly higher in 2018 compared to 2014–2017. The nonparametric Friedman rank sum test showed a statistically significant difference between the mean concentrations of psychrophilic and mesophilic bacteria in Hel, Puck, Gdynia, Sopot and Gdańsk-Brzeźno in 2014–2017 and those in 2018 (χ2 = 5, df = 1, p = 0.025). The results are presented in Table 4.

**Table 4:** The mean and standard deviation of concentration of psychrophilic and mesophilic bacteria (CFU/m^3^) in air over the Bay of Gdańsk in 2018 versus 2014–2017.

Location	Psychrophilic bacteria (CFU/ m^3^)	Psychrophilic bacteria (CFU/ m^3^)	Fold change	Mesophilic bacteria (CFU/ m^3^)	Mesophilic bacteria (CFU/ m^3^)	Fold change
	2018*	2014–2017	2018 vs 2014–2017	2018*	2014–2017	2018 vs 2014–2017
Hel	1.645 × 10^3^ ± 1.273 × 10^3^	1.049 × 10^3^ ± 6.40 × 10^2^	2	1.51 × 10^3^ ± 1.101 × 10^3^	9.00 × 10^2^ ± 6.02 × 10^2^	2
Puck	1.021 × 10^3^ ± 1.95 × 10^2^	9.34 × 10^2^ ± 1.85 × 10^2^	1	5.88 × 10^2^ ± 1.91 × 10^2^	6.07 × 10^2^ ± 2.78 × 10^2^	–
Gdynia	8.11 × 10^2^ ± 1.65 × 10^2^	1.67 × 10^2^ ± 1.17 × 10^2^	5	7.02 × 10^2^ ± 1.63 × 10^2^	1.02 × 10^2^ ± 1.32 × 10^2^; 28 (12–3 × 10^2^)^b^	7
Sopot	5.06 × 10^2^ ± 1.165 × 10^3^; 1.14 × 10^2^ (32–3.386 × 10^3^)^b^	1.27 × 10^2^ ± 5.6 × 10^1^	4	4.86 × 10^2^ ± 1.141 × 10^3^; 9.5 × 10^1^ (2.7 × 10^1^–3.307 × 10^3^)^b^	7.8 × 10^1^ ± 4.8 × 10^1^	6
Gd.-Brzeźno^a^	2.287 × 10^3^ ± 2.677 × 10^3^; 4.89 × 10^2^ (5.5 × 10^1^–5.774 × 10^3^)^b^	4.14 × 10^2^ ± 4.66 × 10^2^; 2.08 × 10^1^ (8.7 × 10^1^–9.47 × 10^2^)^b^	5,5	1.832 × 10^3^ ± 2.104 × 10^3^; 3.94 × 10^2^ (16–4.856 × 10^3^)^b^	3.76 × 10^2^ ± 4.91 × 10^2^; 1.04 × 10^2^ (8.1 × 101–9.43 × 10^2^)^b^	5
p-value^c^	0.025			0.025		

^a^Gd.-Brzeźno—it is an abbreviation of the town name Gdańsk-Brzeźno, ^b^in case of a higher standard deviation than the mean, the median and range (min–max) was calculated, ^c^Friedman test, α-0.05, *The results marked in grey colour have been published in preliminary study^36^.

The concentration of coliforms in the sea spray aerosol (SSA) samples after an emergency discharge of raw sewagewas higher in 2018 than in 2014–2017 at the seaside towns of Hel, Gdynia, Sopot and Gdańsk-Brzeźno. The non-parametric Friedman rank sum test showed that the results were statistically significant (χ^2^ = 5, df = 1, p = 0.025).

Moreover, at the coastal towns of Hel, Puck, Sopot and Gdańsk-Brzeźno statistically significant differences of concentrations of *E. coli,* in the SSA samples from2018 compared to 2014–2017, were detected (χ2 = 4, df = 1, p = 0.046). The mean, median, and standard deviation values for the concentration of coliform bacteria and *E. coli* in SSA are presented in Table 5.

**Table 5:** The mean and standard deviation ofconcentration of coliforms and *E. coli* (CFU/m^3^) in air over the Bay of Gdańsk in 2018 versus 2014–2017.

Location	Coliform bacteria (CFU/m^3^)	Coliform bacteria (CFU/m^3^)	Fold change	*E. coli* (CFU/m^3^)	*E. coli* (CFU/m^3^)	Fold change
	2018*	2014–2017	2018 vs 2014–2017	2018*	2014–2017	2018 vs 2014–2017
Hel	1.98 × 10^2^ ± 2.61 × 10^2^; 1.2 × 10^2^ (0–5.1 × 10^1^)^b^	2 ± 3; 2 (0–1 × 10^1^)^b^	99	2 ± 4; 0 (0–8)	1 ± 2; 0 (0–6)^b^	2
Puck	3.9 × 10^1^ ± 8;	3.5 × 10^1^ ± 1.1 × 10^1^	1	3 ± 5; 0 (0–8)	0.00	3
Gdynia	2 × 10^1^ ± 1 × 10^1^	1 ± 1	20	0.00	0.00	–
Sopot	3 ± 2; 3 (0–7)^b^	1 ± 1	3	1 ± 2; 0 (0–6)	0.00	1
Gd.-Brzeźno^a^	6.7 × 10^1^ ± 6.8 × 10^1^; 5.5 × 10^1^(0–1.49 × 10^2^)^b^	3 ± 1	22	1.8 × 10^1^ ± 3.1 × 10^1^; 7 (0–9.5 × 10^1^)^b^	0.00	18
p-value^c^	0.025			0.046		

^a^Gd.-Brzeźno—it is an abbreviation of the town name Gdańsk-Brzeźno, ^b^in case of a higher standard deviation than the mean, the median and range (min–max) was calculated, ^c^Friedman test, α-0.05, *The results marked in grey colour have been published in preliminary study^36^.

In 2018, in air samples collected in Hel, Sopot and Gdańsk-Brzeźno, a higher concentration of *P. aeruginosa* was detected, but not statistically significant (χ^2^ = 1, df = 1, p = 0.317).

The concentration of *S. aureus* detected in the air in Sopot in 2018, was higher, but not statistically significant (χ^2^ = 2, df = 1, p = 0.157), compared to 2014–2017 (Table 6).

**Table 6:** The mean and standard deviation of concentration of *P. aeruginosa* and *S. aureus* (CFU/m^3^) in air over the Bay of Gdańsk in 2018 versus 2014–2017.

Location	*P. aeruginosa* (CFU/m^3^)	*P. aeruginosa* (CFU/m^3^)	Fold change	*S. aureus* (CFU/m^3^)	*S. aureus* (CFU/m^3^)	Fold change
	2018	2014–2017	2018 vs 2014–2017	2018	2014–2017	2018 vs 2014–2017
Hel	4 × 10^1^ ± 4.7 × 10^1^; 3.2 × 10^1^ (0–9.5 × 10^1^)^b^	1 ± 2; 0 (0–6)^b^	40	6 ± 8; 4 (0–1.6 × 10^1^)^b^	2 ± 6; 0 (0–2 × 10^1^)^b^	3
Puck	0.00	0.00	–	0.00	0.00	–
Gdynia	0.00	5 ± 1.0 × 10^1^; 1 (0–2.2 × 10^1^)^b^	–	0.00	0.00	–
Sopot	2.9 × 10^1^ ± 6.1 × 10^1^; 0 (0–1.67 × 10^2^)^b^	0.00	29	4 ± 7; 0 (0–1.9 × 10^1^)^b^	0.00	4
Gd.-Brzeźno^a^	4.2 × 10^1^ ± 8.5 × 10^1^; 0 (0–2.36 × 10^2^)^b^	0.00	42	0.00	0.00	–
p-value^c^	0.317			0.157		

^a^Gd.-Brzeźno—it is an abbreviation of the town name Gdańsk-Brzeźno, ^b^in case of a higher standard deviation than the mean, the median and range (min–max) was calculated, ^c^Friedman test, α-0.05.

### The relationship between the concentration of bacteria detected in the air samples collected over the Bay of Gdańsk in the 5 coastal towns in 2018 compared to 2014–2017

**Figure 2:**
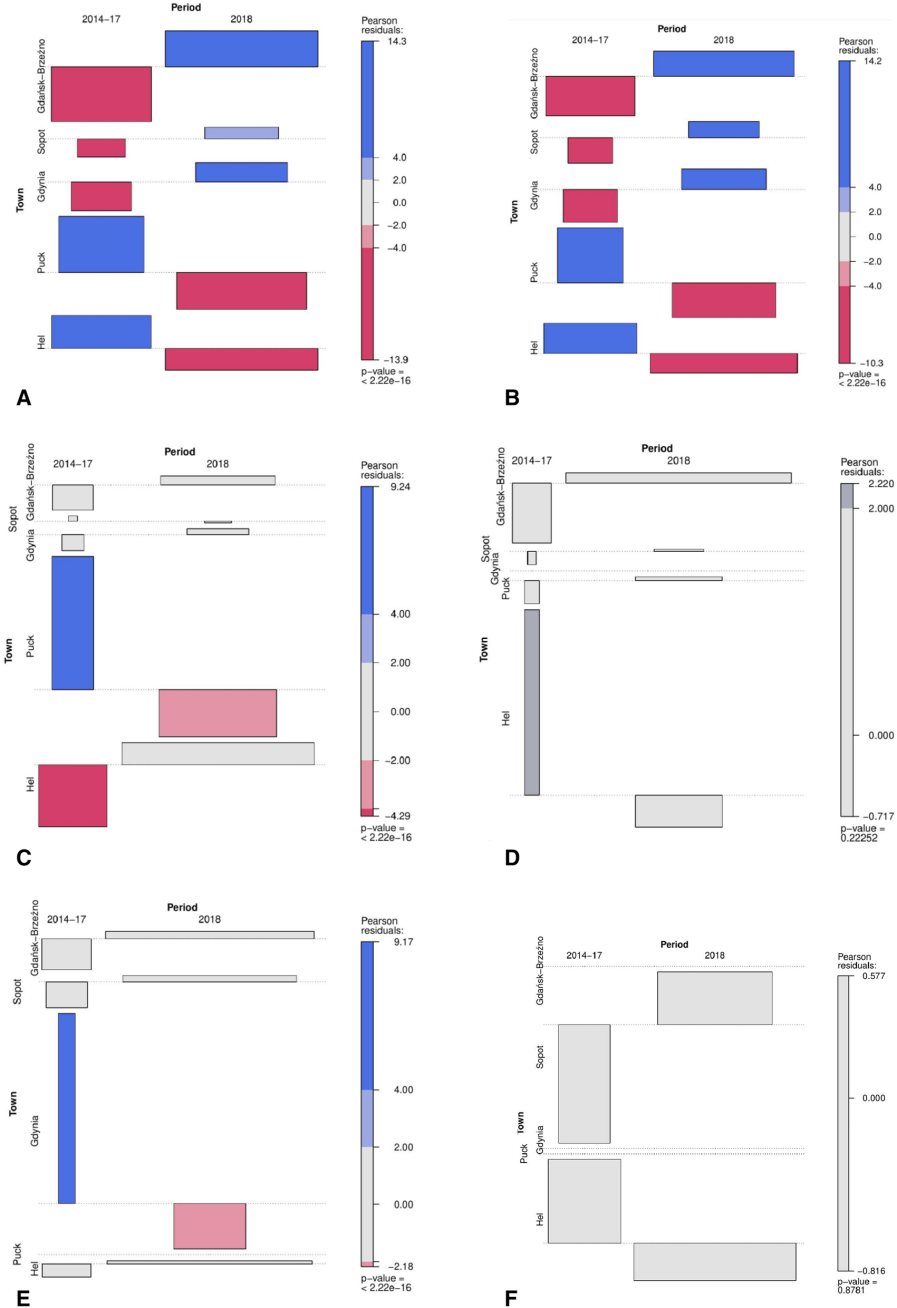
The association of the contingency table with the mean quantity of psychrophilic (**A**) and mesophilic bacteria (**B**), coliform (**C**) and *E. coli* (**D**) bacteria, *P. aeruginosa* (**E**) and *S. aureus* (**F**) bacteria detected in the air samples collected from the Bayof Gdańsk in Hel, Puck, Gdynia, Sopot and Gdańsk-Brzeźno and the time period for the sample collection (2014–2017 and 2018). The blue colour indicates that the observed value is higher than the expected value if the data was random. The red colour specifies that the observed value is lower than expected and the grey colour indicates that the observed value is close to the expected.

The analysis, conducted using contingency tables, made it possible to assess the relationship between the concentration of bacteria detected in the air samples collected over the Bay of Gdańsk in Hel, Puck, Gdynia, Sopot and Gdańsk-Brzeźno in 2018 compared to 2014–2017. Pearson chi-squared test of independence showed that in 2014–2017 in Gdynia, Sopot and Gdańsk-Brzeźno lower than expected concentrations of psychrophilic and mesophilic bacteria were observed, whereas in 2018 it was higher than expected. The result was statistically significant (χ^2^ = 779.51, df = 4, p = < 2.22 × 10^−16^ vs χ^2^ = 681.25, df = 4, p = < 2.22 × 10^−16^) (Fig. 2A, B).

In 2014–2017, the mean concentration of coliforms in Hel was lower than expected, while in 2018 it was very close to the expected value according to the use of the independence test. In 2014–2017 and 2018, in Gdynia, Sopot and Gdańsk-Brzeźno, the concentration of coliforms was very close to the expected value. The result was statistically significant (χ^2^ = 121.87, df = 4, p = < 2.22 × 10^−16^). In 2014–2017 and 2018 it was observed that in Hel, Puck, Gdynia, Sopot and Gdańsk-Brzeźno the mean concentration of *E. coli* was very close to the expected value. The result was not statistically significant (χ^2^ = 5.63, df = 4, p = 0.2225) (Fig. 2C, D).

Also, in2014–2017 and 2018 in Hel, Sopot and Gdańsk-Brzeźno, the mean concentration of the *P. aeruginosa* in the air was close to the expected concentration. The result was statistically significant (χ^2^ = 87.02, df = 4, p = < 2.22 × 10^−16^). In 2014–2017 and 2018 in Hel and Sopot, the concentration of *S. aureus* was very close to the expected concentration according to the use of the independence test. However, the result was not statistically significant (χ^2^ = 1.25, df = 4, p = 0.878) (Fig. 2E, F).

### The effect of meteorological parameters on the concentration of bacteria in the air in 5 coastal towns located on the Bay of Gdańsk

The impact of meteorological parameters (relative humidity, air temperature, wind speed and direction) on the concentration of psychrophilic and mesophilic bacteria, coliforms, *E. coli*, *P. aeruginosa* and *S. aureus* in the air over the Bay Gdańsk in 2018 and 2014–2017 was examined. With the use of Spearman’s correlation a statistically significant positive relationship between the relative humidity of the air over the Bay of Gdańsk and the concentration of psychrophilic bacteria (Rs = 0.368, p = 0.0002) and mesophilic bacteria (Rs = 0.323, p = 0.001) was observed. The results of Spearman’s correlation analysis showed a statistically significant positive relationship between the temperature of air in the Bay of Gdańsk and the concentration of psychrophilic bacteria (Rs = 0.235, p = 0.02), mesophilic bacteria (Rs = 0.198, p = 0.05), coliforms (Rs = 0.489, p = 0.0001), *E. coli* (Rs = 0.289, p = 0.005) and *S. aureus* (Rs = 0.305, p = 0.002). Using Spearman’s correlation, a statistically significant positive relationship was observed between the wind speed measured in 5 coastal towns, and the concentration of psychrophilic bacteria (Rs = 0.301, p = 0.003) and mesophilic bacteria (Rs = 0.3, p = 0.003). The concentration of bacteria in the air over the Bay of Gdańsk in Hel, Puck, Gdynia, Sopot and Gdańsk-Brzeźno was also correlated with the wind direction. The results of the Kruskal–Wallis test showed a statistically positive correlation between the wind direction and the concentration of psychrophilic bacteria (χ^2^ = 53.992, df = 16, p = 5.201 × 10^−6^) and mesophilic bacteria (χ^2^ = 50.706, df = 16, p = 1.768 × 10^−5^). Moreover, a statistically significant positive relationship was observed between the wind direction and the concentration of coliforms (χ^2^ = 39.697, df = 16, p = 0.0008), *P. aeruginosa* (χ^2^ = 25.275, df = 16, p = 0.065) and *S. aureus* (χ^2^ = 39.697, df = 16, p = 0.0008).

### Bacterial emission from the sea-surface microlayer to atmospheric air in 5 coastal towns on the Bay of Gdańsk

We assessed whether SML emissions led to bacterial enrichment of the air in the coastal towns of Hel, Puck Gdynia, Sopot and Gdańsk-Brzeźno. Based on the two-sample.

t-test, in 2018 higher enrichment values were found with respect to psychrophilic bacteria in Sopot and Gdańsk-Brzeźno compared to 2014–2017. The results were positively correlated but were not, statistically significant (p = 0.573). The maximum enrichment factor for aerosolized psychrophilic bacteria in Sopot was 9 in 2018, compared to 0 in 2014–2017. The maximum enrichment factor for psychrophilic bacteria in Gdańsk-Brzeźno was 4 in 2018 compared to 2 in 2014–2017. A greater enrichment factor of mesophilic bacteria was also observed in the air in the above-mentioned cities; however, this was not statistically significant (p = 0.623). The maximum enrichment factor for mesophilic bacteria was found in 2018 compared to 2014–2017, respectively, in Sopot (18) vs (1) and Gdańsk-Brzeźno (3) vs (2). Higher, but not statistically significant (p = 0.419) enrichment factors in coliform bacteria were found in 2018 compared to the analysis carried out in 2014–2017 , respectively, in Hel (768) vs (4), Gdynia (11) vs (1), Sopot (24) vs (2) and Gdańsk-Brzeźno (80) vs (15). The maximum enrichment factor for *E. coli* in Hel was 102 in 2018, compared to 33 in 2014–2017 and in Gdańsk-Brzeźno was 73 in 2018, compared to 0 in 2014–2017 but not statistically significant (p = 0.1). The maximum enrichment factor for *P. aeruginosa* in Sopot was 61 in 2018, without being statistically significant on the Wilcoxon rank sum test with continuity correction (p = 1). The maximum air enrichment factor for *S. aureus* in Gdańsk-Brzeźno was 8 in 2018, compared to 3 in 2014–2017. The result was positively correlated (p = 0.033).

## Discussion

Samples collected in 2018 after an emergency discharge of sewage to the Bay of Gdańsk showed a statistically significant, higher concentration of psychrophilic and mesophilic bacteria in the sea-surface microlayer and air over the Bay of Gdańsk (Hel, Sopot, Gdańsk-Brzeźno) compared to 2014–2017. In 2018, a greater concentration of coliforms wasdetected in water samples collected from the Bay of Gdańsk in Puck, Sopot and Gdańsk-Brzeźno. In addition, a higher concentration of *E. coli* was found in water samples collected in Puck and Gdańsk-Brzeźno. In 2018, a significantly greater concentration of coliforms was detected in the air in Hel, Puck, Gdynia, Sopot and Gdańsk-Brzeźno compared to 2014–2017. Moreover in 2018, in Hel, Puck, Sopot and Gdańsk-Brzeźno, a statistically significant, higher concentration of *E. coli* was detected in the air. Pearson’s chi-squared test and the multidimensional data analysis, conducted using contingency tables, showed a statistically significant positive correlation between the concentration of psychrophilic, mesophilic and coliform bacteria in the water and air in 2018 compared to 2014–2017. These results suggest that the increased concentration of mesophilic bacteria, coliforms and *E. coli* in the seawater of the Bay of Gdańsk and in the coastal air could have been the result of an emergency discharge of raw sewage in 2018. For example, in Kuwait, after a failure at the Mishref sewage pumping station, a greater concentration of coliforms, *E. coli* and faecal streptococci was detected in the waters of Kuwait Bay^29^. The authors showed that the raw sewage discharged for 3 years (2009–2012), directly into the bay, bacteriologically contaminated approximately 20 km of the coast^29^. In another study, Poté et al. (2009) have found that rainwater and sewage flowing out of an underwater collector at a depth of 30 m had the greatest impact on the pollution of the waters of the Bay of Vidy, Lake Geneva, Switzerland^28^.

An attempt was made at the next stage of our research to assess the impact of temperature, humidity, wind speed and wind direction on the presence of bacteria in the air of the coastal towns of Hel, Puck, Gdynia, Sopot and Gdańsk-Brzeźno, after an emergency discharge of sewage into the Bay of Gdańsk. There was a positive relationship between airborne bacteria (psychrophilic andmesophilic bacteria, coliforms, *E. coli, S. aureus*) and the air temperature. It is possible that UV radiation can quickly destroy airborne bacteria. However, the outdoor atmospheric bacteria can tolerate extreme sunlight and moderately high temperatures due to their spore form and pigments^40–42^. According to *Aller *et al. (2005) some bacteria and viruses are likely embedded in transparent gel-like organic particles that can provide some degree of physical protection against UV radiation and drying^43^.

Moreover, there wasa statistically significant relationship between relative humidity and the concentration of psychrophilic and mesophilic bacteria in coastal towns. Recent studies of bioaerosols by other authors have also shown that the relative humidity of the air can affect the species composition of microorganisms and their survival in the air^44,45^. Brągoszewska and Pastuszka showed thathigh relative humidity may result in cell clumping, which possibly increases the odds of microorganism survival^45^. Our study showed a statistically significant, positive relation between a higher concentration of psychrophilic and mesophilic bacteria, coliforms and *S. aureus* and the wind blowing from the north and north-east (i.e. from the sea towards the land). In microbiological air tests conducted by *Montero *et al. (2016) along the quay in Flushing Bay, Queens, New York (USA), correlations were found between the concentration of microorganisms in the coastal air and the wind direction. The total concentration of bacteria detected in air samples taken along the coast was higher when the wind was blowing from the sea. When the wind was blowing from the land, a higher concentration of fungal mould spores was detected^46,47^. Our study indicates a statistically significant relationship between the wind speed and the concentration of psychrophilic and mesophilic bacteria after an emergency discharge of sewage into the Bay of Gdańsk. The results of our team's research have shown for the first time ever that air velocity plays a key role in bacterial emissions from marine coastal water of the Bay of Gdańsk to coastal air^48–50^. What's more, it has also been shown that the process of breaking wind waves is also influenced by wind speed, which causes the formation of bubbles in seawater^48,51^. The bubbles selectively accumulate hydrophobic matter and microorganisms, which are transported towards the water surface and are partially emitted into the air [^51–55^]. For example *Uetake *et al*.* showed that the wind speed was positively correlated with wave height in Tokyo Bay. High wind speeds above the bay's surface correlated with greater aerosol production by the bubble bursting process during breaking waves, especially on the shore^55^. Other researchers found thatthe viable and total bacterial concentrations was significantly higher when the wind speed was greater than 5.4 m s^−1^ (exceeding a Beaufort force of 3, at which wave breaking can occur)^53^. *Dukier *et al. also showed a positive correlation between wind speed and the amount of bacteria and viruses in the coastal air along the urban wharf at Louis Valentino Pier in the harbour of Brooklyn and Flushing Bay (FB), Queens, New York, (USA)^5^. Interesting observations were also presented by*Rahlff *et al., they showed that enrichment of bacteria in the SML occurred solely at a U_10_ wind speed of ≤ 5.6 m s^−1^ in the tunnel and ≤ 4.1 m s^−1^ in the Baltic Sea^56^.

And finally, our study showed a clear tendency of SSAenrichment with psychrophilic, mesophilic bacteria, and *E. coli* and *P. aeruginosa* in Sopot and Gdańsk-Brzeźno. The high, although not statistically significant, factor of air enrichment with coliforms was detected in 2018 in Hel, Gdynia, Gdańsk-Brzeźno and Sopot. The high factor of air enrichment with mesophilic bacteria, coliforms, *E. coli*, *P. aeruginosa* or *S. aureus* may be associated with the emergency discharge of wastewater into the Motława River flowing into the Bay of Gdańsk. Therefore, it is suggested that in the event of a malfunction of a sewage treatment plant, as well as after floods or sudden rainfall, the public should be informed about the sanitary and epidemiological status of the coastal waters and be recommended to limit their use of coastal leisure areas.

In our previous studies, at the mouth of the Vistula River, we showed a 12-fold greater enrichment rate in SSA for mesophilic, potentially pathogenic bacteria compared to psychrophilic bacteria^48^. We also provided evidence that oxygen supersaturation in the surface water may contribute to enhanced bubble-mediated sea-to-air bacteria transport , in particular during the presence of a summer phytoplankton bloom in the Gulf of Gdańsk^48^. Blanchard and Syzdek (1970) were the first to study the phenomenon of enrichment of marine aerosols drops with bacteria. The authors demonstrated that air bubbles breaking at the air–water interface can remove bacteria that concentrate in the surface microlayer and eject them into the atmosphere. The bacterial concentrations in the drops ejected from the bubbles may, depending on the drop size, be from 10 to 1000 times that of the water in which the bubbles burst^54^. In turn, other researchers showed 15–25 fold enrichment in bacteria and viruses during transport from SML into the atmosphere. The data support the idea that the SML is a major source of microorganisms entering the atmosphere from water bodies^43^. It should be kept in mind that studying aerosols in nature is extremely difficult as, confounding factors such as ocean and atmospheric circulation patterns prevent convolution of terrestrial and marine sources of airborne microbes^38,56,57^. The mechanism is based on the mutual attraction of negatively charged bacterial cells by cationic vortexes formed below the bubbles. The scavenging of microorganisms in seawater depends on the bubble’ size, the value of the negative charge of anions accumulated on the outer membranes and the charge of the cations accumulated in the vortex under the bubble^52,58^. Further research is needed, both in the laboratory and marine waters, to understand the "transport" of microorganisms in detail.

### Study limitations

The present study has some limitations that we would like to address. First, the studies presented were based on the assessment of the concentration of bacteria grown on microbiological media (described in the Materials and methods section). Not all live bacteria may grow on the microbiological plates, so the concentration of live bacteria in the surface microlayer and sea spray may have been greatly underestimated. Second, most studies on the microorganisms in the sea-surface microlayer have been conducted in the open ocean and coastal water, but studies from the Bay of Gdańsk after an emergency discharge of raw sewage are not available.

## Materials and methods

### Collection of aerosol and seawater samples

In the years 2014–2018, a total of 408 air samples and 186 sea-surface microlayersamples were collected in 5 coastal towns (Hel, Puck, Gdynia, Sopot and Gdańsk-Brzeźno) in the Bay of Gdansk. In the years 2014–2017, the air samples were collected between the 22nd of May and 22nd of July, every 20 days between 9:00 a.m. and 2:00 p.m. In 2018, after an emergency disposal of raw sewage, the air samples were collected between the 22nd of May and 22nd of July, every 11 days between 9:00 a.m. and 2:00 p.m. Air and water samples were not collected under rainy conditions. The air samples were collected for 10 min by impaction with a SAS Super ISO 100 (Milan, Italy) sampler. The nozzle of the sampler was positioned perpendicularly to the wind direction. The sampler automatically collected 100-L samples of air. The microorganisms passed through small holes in the sampler, directly on to Petri dishes containing an agar medium appropriate for each type of organism. The maximum efficiency of collection is for particulate matter with a d50 = 2–4 μm. The flow rate is 90 lpm. All removable parts of the air sampler were sterilized by autoclaving before sampling and the sterilized sampler head was cleaned between samples with a 70% ethanol solution^17^. The samples of seawater were collected from the microlayer (SML) ≤ 100 μm with a sterile 3 mm thick glass plate sized 50 cm × 50 cm. The plate was immersed in the seawater at an angle of 45 degrees and once the surface of the water was stable the plate was pulled out with a vigorous movement. Water from both sides of the plate was removed with a rubber wiper into sterile glass bottles^59,60^. The samples of water from the sea microlayer were stored in a cooling container at 4 °C and delivered to the laboratory within 4 h from collection for further analysis^36^.

### Microbiological analysis of bioaerosols

The concentration of mesophilic bacteria in the air samples was determined on trypticase soy agar (TSA) by Merck/Germany, after a 24–48-h incubation period at 37 °C. For psychrophilic bacteria culture TSA agar was used. The incubation was carried out at 22 °C for 72 h. The concentration of coliforms and *Escherichia coli* was determined on Chromocult® Coliform Agar by Merck/Germany, after a 24-h incubation period at 37 °C. The concentration of *Pseudomonas aeruginosa* bacteria was determined on Cetrymide agar by Merck/Germany after a 24-h incubation period at 37 °C. The concentration of *Staphylococcus sp*. was determined on Mannitol Salt Agar (MSA) by Merck/Germany. Confirmatory tests involved making Gram stained preparations, performing tests for the presence of aminopeptidase and catalase enzymes, and confirming the ability of the studied bacteria to ferment glucose in anaerobic conditions. *Staphylococcus aureus* was also differentiated from *Staphylococcus epidermis* saprophytic strains with the rabbit plasma test. The concentration of bacteria was expressed as a colony-forming unit (CFU) per 1 m^3^ of air (CFU/m^3^). To calculate the number of microorganisms in the air samples, the Feller measuring table attached to the air sampler manual was used^61^. The concentration of bacterial colonies (CFU/m^3^) was calculated using the equation contained in the publication^17^.

The colonies collected should be revised by the Eq. (1)

1 $${\text{Pr}} = {\text{N}}\left( {{1}/{\text{N}} + {1}/{\text{N}} - {1} + {1}/{\text{N}} - {2} + {1}/{\text{N}} - {\text{r}} + {1}} \right)$$

where Pr is the revised colony in stage, N is the concentration of sieve pores, and r is the concentration of viable particles counted on the agar plate.

The concentration of bacterial colonies (CFU/m^3^) was calculated using the following Eq. (2)

2 $${\text{C}}\left( {{\text{CFU}}/{\text{m3}}} \right) = {\text{ T}} \times {1}000{\text{t}}\left( {{\text{min}}} \right) \times {\text{F}}\left( {{\text{L}}/{\text{min}}} \right)$$

where C – airborne bacteria concentration; CFU – colony-forming unit; T – total colonies after application of the Pr statistical correction; t – sampling time and F – airflow rate.

Enumeration of bacteria in the air was conducted according to the Polish Standard (PN-89 Z-04111/02-Air purity protection, Microbiological testing, Determination concentration of bacteria in the atmospheric air (emission) with sampling by aspiration and sedimentation method 1989)^62^. We also used theNIOSH Manual of Analytical Methods (2003-154)—Method Number 0-2000 and the general provisions for EPA-Sampling, Laboratory, and Data Considerations for Microbial Data Collected in the Field^63^.

### Microbiological analysis of sea-surface microlayer

The overall concentration of psychrophilic and mesophilic bacteria was determined with the pour plate method after incubation of 1 ml of seawater on tryptic soy agar from Merck (Germany). The results were read after 72 h and 48 h of incubation at 22 °C and 37 °C. The colony-forming unit count was used to determine the concentration of bacteria in 1 ml seawater samples (CFU/ml). The determined pour plate method and membrane filtration method were used to study the water samples, according to the Polish Standard PN-ISO 6222^64^ and to the American Public Health Association standard^65^. Water samples (SML) of 100 ml each were filtrated through sterile filters (pore size 0.45 μm) and then placed on selective agar mediums. The concentration of coliform bacteria was estimated on Chromocult® Coliform Agar by Merck/Germany after 24-h of incubation at 37 °C, according to the Polish Standard PN-EN ISO 9308-1:2014-12^66^. The *P. aeruginosa* bacteria concentration was determined on Cetrymide Agar by Merck/Germany after 24-h of incubation at 37 °C, according to the Polish Standard PN-EN ISO 16266:2009^67^. The concentration of *Staphylococcus sp*. was determined on MSA by Merck (Germany) according to the standard developed by the Polish Hygiene Institute PB-01-PM-NIZP-PZH ZHK:2007^68^. The colony-forming unit (CFU/ml) was used to determine the concentration of bacteria in 100 ml of SML. Gram-stained preparations were made. Tests for the presence of aminopeptidase and catalase enzymes and confirmation of the ability of the studied bacteria to ferment glucose under anaerobic conditions were performed. *S. aureus* was differentiated from *S. epidermis* by a tube coagulase test^36^. Microbiological research of seawater in coastal bathing areas was carried out in accordance with the Regulation of the Minister of Health of January 17, 2019 on the supervision of the quality of water in bathing areas and occasional recreational places (Dz. U. z 2019, poz. 255), the Bathing Water Directive (Directive 76/160/EEC 2006) and Water Law (Journal of Laws of 2018, item 2268, as amended)^69–71^.

### Characterization of meteorological conditions

During the collection of samples between spring and summer during 2014–2017 and in 2018, air temperature, humidity, wind speed and wind direction were recorded using a GMH 3330 thermo-hygrometer by Greisinger (Germany). The air temperature during 2014–2017 ranged from 3 °C to26°C (spring season), and from 16 to 20 °C (summer season). Relative humidity during spring was between 30 and 88%, and from 59 to 82% during summer . Wind speed during spring varied between 0 and 31 km h^−1^, and between 7 and 25 km h^−1^ in the summer. The air temperature in 2018 fluctuated between 1 and 27 °C in the spring, and between 15 and 27 °C during summer. Relative humidity in the spring season ranged between 39 and 93%, and from 44 to 70% in the summer season. Wind speed during spring varied between 0 and 15 km h^−1^, and between 2.6 and 32 km h^−1^ in the summer^36^.

### Model evaluation of bacterial emission from SML to atmospheric air

In order to assess bacterial emission from seawater to atmospheric air we calculated the enrichment factor (EF) according to the following formula^72,73^:

$${\text{EF}} = {\text{L}}_{{\text{A}}} /{\text{L}}_{{\text{W}}}$$

where L_A_—oncentration of bacteria in sea aerosols (CFU/1 ml), L_W_—concentration of bacteria in the sea surface microlayer in the Bay of Gdańsk (CFU/1 ml).

The concentration of bacteria in marine aerosol (L_A_) was calculated from the formula:

$${\text{L}}_{{\text{A}}} = {\text{L}}/{\text{A }}\left( {{\text{ml}}} \right)$$

where L—concentration of bacteria in the air (CFU/m^3^), A—volume of sea derived droplets in air for a given wind speed u(10), A = exp((-2.62 + 0.59 u(10)) 0.142857 (ml/m^3^).

Where: 0.142857 = (1000/7)10–3, 7‰ is the mean salinity of the Baltic Sea within (7–8‰), the value of 7 ‰ salinity fits to the range of calculations considered in our model, while 10–3 results from the conversion of µg/ml, assuming the density of water at 1 g/ml.

u(10) is the wind speed in m/s measured 10 m above the surface of water, under the conditions of a wind fetch over the sea of at least 5–10 km.

### Statistical analysis

A Pearson’s chi-squared test for independence was performed to assess significant differences between the categorical variable: for one “sample collection years” (two subgroups; 2014–2017 and 2018), and for the second categorical variable:“place” (five subgroups: Hel, Puck, Gdynia, Sopot and Gdańsk-Brzeźno). The contingency tables obtained this way are presented in the form of association figures with the values of Pearson’s residuals and the p value of the independence test^74^. Association plots visualize the table of Pearson residuals: each cell is represented by a rectangle that has a height proportional to the corresponding Pearson residual and width proportional to the square root of the expected value. Thus, the area is proportional to the raw residuals. The rectangles representing each cell in the table are positioned relative to a line representing independence. Cells with an observed frequency greater than expected are shown above the line and cells with an observed frequency lower than expected are shown below the line^75^.

In each of the association figures, the blue colour means that there are more observations than would be expected if the data was random. Negative Pearson residuals (the red colour) means that the cell values were smaller than expected. The grey colour represents the data where the concentrations are close to the expected, i.e., the null hypothesis of the independence test is true. To assess the differences between the mean concentration of bacteria detected in seawater and air in the two time periods, 2014–2017 and in 2018, the non-parametric Friedman rank sum test was used. To assess the differences between bacterial aerosol concentrations and meteorological parameters, the Kruskal–Wallis ANOVA non-parametric test was used, and the Spearman correlation was determined. In order to compare the bacterial enrichment of the seawater to air between 2014–2017 and 2018, we used the following tests: a two-sample t-test (where the Shapiro–Wilk test showed that the compared groups are from a normal distribution population) and the Wilcoxon rank sum test with continuity correction (for all other cases). The statistical significance of the differences between the groups was set at p < 0.05. Statistical analysis of the results was carried out using software R (2018)^76^.
